# Ovarian Dropsy

**Published:** 1856-09

**Authors:** J. T. Jameson

**Affiliations:** South Otselic, Chenango Co., N. Y.


### ART. V.—*Ovarian Dropsy*. By J. T. Jameson, M. D., South Otselic, Chenango Co., N. Y.

I am not, Mr. Editor, about to narrate any great exploit of my own in relation to this Ovarian Cyst, as it was judged to be, but simply to communicate to your readers a plain unvarnished tale of nature's handiwork in respect to the same, with the hope that the recital may not be altogether uninteresting or unprofitable to by professional brethren.

March 8th, I was requested to visit Mrs. G., whom I had attended a fortnight before in her second confinement, which was perfectly natural and easy. I was informed that she appeared to be doing well for the first two or three days, the lochia being rather more than usually copious; but was then seized with cold chills, followed by heat and copious perspiration, pain in the head, and tenderness of the abdomen, for which she was doctored by the women folks in attendance.

*Present State.* Countenance a little flushed; tongue considerably coated; thirst; no appetite; skin a little hotter than usual; pulse about 120, and small; severe darting pains in the head occasionally; soreness and stiffness of the neck; noise in the ears; a slight noise distresses her; is easily excited, and a slight disturbance causes palpitation; sleep disturbed by frightful dreams, and frequently awakes as if in terror, accompanied by palpitation; breaks out into a profuse perspiration on falling into a dose; feels very weak, but sits up about two hours daily; milk almost gone; bowels rather inclined to constipation; slight lochial discharge at present.

I was disposed to refer some of the above unfavorable symptoms to close confinement in an over-heated room. On my entering the apartment, I found it quite oppressive, and communicating a very unpleasant odor, owing, as I judged, to the pent up vitiated atmosphere which filled it. The fire was reduced, door opened, and bed-clothes diminished, by which in a couple of hours she felt relieved, the skin being cooler, pulse less frequent, and headache diminished.

To take a pill of pil. hydrarg., extr. hyoscy., and morphine each night at bed time, a laxative as the bowels may require, and a mixture of infus. calumb., sod. bi carb., spt. eth. nitros., and spt. am. aromat., three times a day. Lemonade for drink; light diet; more pure air.

Under the use of these means, the above unpleasant symptoms disappeared, leaving, however, considerable debility. Very soon she began to complain of distressing symptoms in relation to the pelvic organs, on account of which I was again desired to see her, and found the patient suffering from darting pains through the pelvis; bearing down sensation; difficulty and pain in making water, with frequent desire to do so; pain about the anus, especially when the bowels are moved; pain down the thighs; the bearing down feeling is so distressing in the erect position that she is almost constantly recumbent; tongue a little coated, but no fever of consequence; bowels rather constipated.

On examination per vaginam, the patient standing, there did not appear to be any prolapsus uteri; the vagina seemed somewhat tender, but not particularly hot, and the os and cervix uteri did not project into it at all. I did not at this time detect any tumor. Some of the old feminines were disposed to visit the patient's troubles upon my head, as having transgressed either by omission or commission, and from this time until the 25th of July, I saw very little of the patient, but understood that a variety of quack medicines were resorted to without any benefit—that the unpleasant symptoms connected with the pelvic organs continued, rendering the recumbent posture almost constantly necessary, and that the patient emaciated. About the last of June, the patient perceived a tender spot in the right iliac region, and, on further examination, distinguished a tumor. Dr. Whitford was sent for, who pronounced the swelling to be ovarian, ordering iodine and mercurial ointment to be applied.

In the night of the 25th of July, I was sent for in haste, the patient being reported as dying, and found her suffering from severe pain, in the lower part of the abdomen, of a distressing bearing down character, and acute pain over the sacrum. On inspection of the abdomen, I found a large tumor occupying the right iliac region, and extending about as high as the umbilicus, and as far as the median line of the abdomen; at its upper border, it was well defined, whilst on each side of it was not sa, and, below, appeared to dip behind Poupart's ligament into the pelvis. I examined per vaginam, and found my finger obstructed, not far from its entrance, by a tumor projecting from its posterior wall, the contents of which were manifestly fluid; pressure on this swelling caused some pain, and on passing the finger upward between it and the neck of the bladder, I was enabled to feel the os uteri. This tumor in the vagina appeared to me to be the chief cause of her distress, I, therefore, punctured it with a lancet, when, perhaps, a teacupful of serous fluid escaped, the patient exclaiming that she was easy, and that the fluid discharged felt hot. There appeared to be no diminution in the size of the abdominal tumor. She soon fell asleep.

I left a little morphine.

26th. She was seen by Dr. W., who ordered, as he subsequently informed me, pills of pil. hydr. and pulv. Doveri, and a mixture of spt. eth. nitros. and tinct. camph. co. Vinegar and salt to be applied to the abdomen, which was very tender over the tumor.

27th. I met Dr. W. for the first time. The tumor was very distinct on external examination of the abdomen, and evidently contains fluid; tenderness on pressure not so great as yesterday; on examination per vaginam, I perceived a slight fullness in the situation of the puncture made in the night of the 25th, and high up the tumor in the abdomen could be perceived, but more distinctly still by examination per anum. The patient feels comfortable, and has experienced but little pain since the night of the 25th. It was deemed best to wait, and watch the course of events.

Cont. Med., 28th. On our meeting next day at 2, P. M., we were informed that at 10, A. M., there had taken place a sudden and copious discharge of serous fluid per vaginam, and which continues somewhat at present. The fluid being collected on cloths, it is impossible to state the quantity, but from the number of cloths, it must have been considerable. The abdominal tumor has almost disappeared, the abdomen now feeling quite soft, where, yesterday it was hard and tense, as well as quite tender to the touch, and considerable tenderness still remains; appears comfortable.

Cont. Med., ol. ricini.

29th. Whilst on the *pot de-chambre*, last night, there was another discharge of fluid, the abdomen being still softer, and tumor less evident.

Cont. Med. From this time my attendance ceased, Dr. W. continuing to minister to the patient; but I learned, that under the use of alteratives and diuretics, there was a gradual amendment, and restoration to a good state of health. It is now some years since the event. I have been in the habit of seeing the patient at intervals, and, when last seen, there had been no return of the disease.

*Remarks.*

1. It appears probable that the swelling in the above case was a cyst connected with the right ovary, and that the vascular excitement or inflammation which manifestly existed about the pelvic organs soon after delivery, either gave rise to the development of the said cyst, or, if already existing, as I am inclined to suppose, stimulated it to greater activity.

Whilst inspecting, some time ago, the body of a female who died of erysipelatous peritonitis, I observed a cyst about the size of a hen's egg, connected with the left ovary, containing a transparent fluid, and its internal membrane very vascular. Now, it is not unreasonable to suppose that some such cyst might have existed for some time in the ovary of Mrs. G., and, as stated above, been roused to active development by the vascular excitement existing in the pelvic viscera, the uterus more especially. I ought to have previously stated that an interval of about seven years had elapsed between Mrs. G.'s first and second pregnancy, and that her health during this period had been generally good.

2. What was the nature of the swelling in the vagina punctured by me in the night of the 25th of July? Was it connected with the abdominal tumor, and if so, why was not this evacuated, or was it only a serous effusion into the submucous cellular tissue of the vagina? It would have been interesting, if possible, to have determined the precise place of giving way of the cyst, whether at the seat of puncture or not.

